# Pharmacogenetic Analysis of Voriconazole Treatment in Children

**DOI:** 10.3390/pharmaceutics14061289

**Published:** 2022-06-17

**Authors:** Romy Tilen, Paolo Paioni, Aljoscha N. Goetschi, Roland Goers, Isabell Seibert, Daniel Müller, Julia A. Bielicki, Christoph Berger, Stefanie D. Krämer, Henriette E. Meyer zu Schwabedissen

**Affiliations:** 1 Division of Infectious Diseases and Hospital Epidemiology, University Children’s Hospital Zurich, Steinwiesstrasse 75, 8032 Zurich, Switzerland; paolo.paioni@kispi.uzh.ch (P.P.); christoph.berger@kispi.uzh.ch (C.B.); 2 Biopharmacy, Department of Pharmaceutical Sciences, University Basel, Klingelbergstrasse 50, 4056 Basel, Switzerland; roland.goers@unibas.ch (R.G.); isabell.seibert@unibas.ch (I.S.); 3 Biopharmacy, Institute of Pharmaceutical Sciences, Department of Chemistry and Applied Biosciences, ETH Zurich, Vladimir-Prelog-Weg 4, 8093 Zurich, Switzerland; goetscha@student.ethz.ch (A.N.G.); stefanie.kraemer@pharma.ethz.ch (S.D.K.); 4 Institute of Clinical Chemistry, University Hospital Zurich, Rämistr. 100, 8091 Zurich, Switzerland; daniel.mueller@usz.ch; 5 Paediatric Research Centre, University Children’s Hospital Basel, Basel, Spitalstrasse 33, 4056 Basel, Switzerland; juliaanna.bielicki@ukbb.ch

**Keywords:** children, pediatric pharmacology, voriconazole, therapeutic drug monitoring, pharmacogenetics, non-linear mixed effects modelling, CYP2C19, CYP3A4, ABCC2, ABCG2

## Abstract

Voriconazole is among the first-line antifungal drugs to treat invasive fungal infections in children and known for its pronounced inter- and intraindividual pharmacokinetic variability. Polymorphisms in genes involved in the metabolism and transport of voriconazole are thought to influence serum concentrations and eventually the therapeutic outcome. To investigate the impact of these genetic variants and other covariates on voriconazole trough concentrations, we performed a retrospective data analysis, where we used medication data from 36 children suffering from invasive fungal infections treated with voriconazole. Data were extracted from clinical information systems with the new infrastructure *SwissPK*^cdw^, and linear mixed effects modelling was performed using R. Samples from 23 children were available for DNA extraction, from which 12 selected polymorphism were genotyped by real-time PCR. 192 (49.1%) of 391 trough serum concentrations measured were outside the recommended range. Voriconazole trough concentrations were influenced by polymorphisms within the metabolizing enzymes CYP2C19 and CYP3A4, and within the drug transporters ABCC2 and ABCG2, as well as by the co-medications ciprofloxacin, levetiracetam, and propranolol. In order to prescribe an optimal drug dosage, pre-emptive pharmacogenetic testing and careful consideration of co-medications in addition to therapeutic drug monitoring might improve voriconazole treatment outcome of children with invasive fungal infections.

## 1. Introduction

Invasive fungal infection (IFI) is a serious infectious complication and a major cause of morbidity and mortality in neonates and in children with primary or acquired immunodeficiency [1]. Voriconazole is among the most important and recommended antifungal drugs to treat IFIs in children >2 years of age [2,3]. It is also prescribed (off-label) in children younger than 2 years of age although optimal dosing for this age category has not yet been established [4,5]. Voriconazole is an antifungal triazole with a broad-spectrum activity against yeasts and opportunistic molds, particularly *Aspergillus* spp. [6,7]. Since, invasive aspergillosis, the main indication of voriconazole in children, is associated with a high mortality rate (>50%), an efficacious and safe therapy is needed [8]. Serum trough concentrations (*C*_trough_) of 1 to 5.5 mg/L are considered adequate for treatment [9,10,11,12], while *C*_trough_ of <1 mg/L were associated with increased mortality in children in some [13], but not in other studies [14]. Different studies showed improved efficacy in IFIs when voriconazole *C*_trough_ is ≥ 1 mg/L [15], but the impact of supra-therapeutic voriconazole *C*_trough_ > 5.5 mg/L and the risk for adverse reactions or toxicity remains uncertain [16,17,18]. Routine therapeutic drug monitoring (TDM) is strongly recommended [15,19], as targeting the narrow therapeutic window of *C*_trough_ between 1 mg/L and 5.5 mg/L is challenged by the high inter- and intraindividual pharmacokinetic variability in children [20,21]. One cause of this variability is a saturable metabolic clearance, which leads to a dose-dependent elimination half-life and non-linear pharmacokinetics. This effect was shown in adults at regular doses (4 mg/kg q12h) and in children at higher doses (8 mg/kg q12h), the latter corresponding to the recommended maintenance dose in children from 2 to 12 years of age [22,23,24]. Voriconazole is metabolized in vitro by the cytochrome P450 enzymes CYP2C19, CYP2C9, and CYP3A4, while in vivo studies suggested a major role of CYP2C19 in the enzymatic conversion of voriconazole to voriconazole N-oxide [3]. The later finding with predominance of an individual metabolizing enzyme in combination with the narrow therapeutic window results in a considerable risk for drug-drug interactions. Taken together, the pharmacokinetic profile of voriconazole bears a high risk for treatment failure or toxicity. The target range of *C*_trough_ is often not reached [25,26], even if recommended empirical maintenance doses are administered in children, according to their age and body weight (https://db.swisspeddose.ch/voriconazole, accessed on 18 November 2021).

Multiple factors affect the pharmacokinetic variability of voriconazole in pediatric patients [5]. These include the aforementioned non-linear kinetics and the potential for drug-drug interactions in addition to patient-specific characteristics such as age, body weight, liver function, and the CYP2C19 genotype. Except for CYP2C19 [27,28], only few studies have been performed in children investigating the influence of pharmacogenetics (PGx) on the pharmacokinetics of voriconazole [29,30]. One such study by Allegra et al. has recently shown that in addition to CYP2C19, single nucleotide polymorphisms (SNPs) in other genes involved in the metabolism and transport of voriconazole influence *C*_trough_ [30]. Accordingly, it is essential to understand how genetics affect the pharmacokinetics, efficacy and safety of voriconazole in children in order to adjust drug therapy and achieve treatment success. Therefore, our aim was to investigate the impact of variants in genes potentially involved in voriconazole metabolism or transport and of typical co-medications on voriconazole *C*_trough_ in children. Understanding the factors affecting voriconazole pharmacokinetics in children is a prerequisite towards individualized dosing schemes to eventually improve the treatment outcome in children suffering from IFIs.

## 2. Materials and Methods

We performed a retrospective data analysis with health-related personal data and in particular data on voriconazole medication including *C*_trough_, information on co-medication and biological material from children with proven, probable or suspected IFI treated with voriconazole and at least one measured *C*_trough_ between 2014 and 2019 in the University Children’s Hospitals Zurich and Basel. The study was approved by the Ethics Committee of the Kanton Zurich, Switzerland (BASEC 2020-00217).

### 2.1. Data Collection and TDM

Thirty-six patients treated in the two children’s hospitals matched the aforementioned inclusion criteria. Consent on use of health-related data and biological material (signed by a legal representative (parent) or by patient if older than 14 years of age) was available from all included patients. Data were extracted from the clinical and laboratory information systems of the hospitals with the recently built Swiss Personalized Health Network (SPHN) platform *SwissPK*^cdw^, a clinical data warehouse designed for the gathering and analysis of routine clinical and study related data for pharmacokinetics analyses under secure conditions [31].

Blood samples were taken immediately before drug application (*C*_trough_) under steady-state conditions. Voriconazole *C*_trough_ values from the University Children’s hospital Zurich were measured in the Institute of Clinical Chemistry of the University Hospital Zurich by liquid chromatography coupled to tandem mass spectrometry (LC-MS/MS) using the commercially available TDM A kit (MassTox^®^, Chromosystems, Munich, Germany). The laboratory of the University Hospital Basel determined the voriconazole *C*_trough_ values of patients from the University Children’s Hospital Basel by a LC-MS/MS inhouse method applying turbulent-flow online extraction (Cyclone^®^, Thermo Fisher Scientific, Reinach, Switzerland) and reversed-phase chromatography (Synergi 4 μm Max^®^, Phenomenex, Basel, Switzerland) after protein precipitation of the serum samples with acetonitrile. For quantification, commercially available calibrators and controls were used (Recipe, Munich, Germany). After validation of the collected health-related data including the voriconazole *C*_trough_ and required corrections based on hospital records, we performed data analysis on the *SwissPK*^cdw^ platform.

### 2.2. Genotyping

From 23 of the 36 children biological material (full blood, viable cells or DNA samples) was retrieved and pseudonymized samples were sent to the laboratory of the Biopharmacy at the Department of Pharmaceutical Sciences at the University of Basel for genetic analysis. In patients undergoing stem cell transplantation the biological material was collected prior to transplantation. Cell samples were used for DNA extraction using the QIAmp DNA Blood Mini kit and the QIAcube (Qiagen AG, Hombrechtikon, Switzerland). DNA samples were analyzed using the Tecan Infinite Pro 200 and the NanoQuantTM plate (Tecan, Maennedorf, Switzerland). Here, the concentration and 260/280-ratio was set to >5 ng/μL and 1.8–2.10, respectively, making the samples eligible for further analyses. Each DNA sample in this study reached this quality.

The DNA was used for determination of the genetic variants as listed in Table 1. For analysis commercial chemistry was used. Briefly, 5 μL TaqMan^®^ Genotyping Master Mix were combined with 0.5 or 0.25 μL of the respective primer/probe mix (Assay) and 1 μL of the respective DNA or positive control. The final reaction volume was 10 μL, with water supplemented. For genotyping reactions with known low minor allele frequency (MAF), namely for detection of rs28399504, rs4986893, rs35599367, rs10264272, and rs776746, a heterozygous control was generated. This control was a mixture of plasmids containing the amplicon either of the reference or of the variant of the respective polymorphism. The respective genotyping polymerase chain reaction was performed and the amplicons were ligated into pDrive (Qiagen PCR cloning Kit), after amplification in *E. coli*, the isolated plasmids were sequenced (Microsynth, Balgach, Switzerland) and therefore verified. After combining the two plasmids, the heterozygous control was used at a concentration of 20 ng/μL in each genotyping run. We gathered the genetic variants for voriconazole based on literature and comparison with information on PharmGKB (https://www.pharmgkb.org/, accessed on 7 May 2021) [29,30,32]. The genetic panel consisted of 12 selected SNPs within the genes of metabolizing enzymes *CYP2C19*, *CYP3A4*, and *CYP3A5* and of drug transporters *ABCC2*, *ABCG2*, and *SLCO1B3* (Table 1).

Following the protocol for secure data transfer previously developed, the genetic information of each individual was encrypted and transferred via the BioMedIT node SciCORE (University of Basel) to the *SwissPK*^cdw^ which is hosted at the BioMedIT node Leonhard Med (ETH Zurich) [33].

### 2.3. Data Analysis by Linear Mixed Effects Modelling

Data were analyzed by linear mixed effects modelling with the package lmer (v1.1-28) in the statistical computing language R (v4.0.4) [34]. Concentrations below the lower limit of quantification (LLOQ, 0.1 mg/L) were set to 0.05 mg/L. The principle model for analysis was ln(*C*_trough_) ~ ln(dose/body weight) + covariates + (1|ID). Covariates were expressed as the difference between a logarithmic (ln) value and a logarithmic (ln) reference value as indicated, as a factor (sex, route of administration, diagnose), or as a numeric value. The numeric values for co-medication were 0 (the drug was not co-administered within the same day) and 1 (the drug was co-administered within the same day); the numeric values for variant alleles of a particular gene were 0 (reference genotype), 1 (heterozygous) or 2 (homozygous for the genetic variants). The subject’s pseudo-identity (ID) was used as the grouping factor with random intercepts. For model building, patient body weight, body surface area, body height, age, sex and secondary diagnose (IFI) as well as route of administration were evaluated as potential covariates first. The respective residues (ln(observed *C*_trough_) − n(predicted *C*_trough_)) were plotted against co-medications and genetic variants. Co-medications and genetic variants with suspected differences in the residues were evaluated as covariates in the model. Finally, interactions were tested between drugs and genes, based on the further inspection of the residues plotted against co-medications and variant genes. Covariates were included if the respective *p* value for the effect (or interaction) was <0.05 and if −2 × log likelihood (−2LL) was significantly reduced (difference in −2LL > 3.84 for one additional degree of freedom). The model was built by alternating between addition and removal of a covariate or interaction.

## 3. Results

### 3.1. Population Description and TDM

In this analysis we included 36 pediatric patients, aged between 0.5 months and 17 years, with proven, probable or suspected IFI, and treated with voriconazole. These patients had a total of 18 different main diagnoses related to primary or acquired immunodeficiency, ranging from different types of leukemia/lymphoma and anaemia to cystic fibrosis. The characteristics of the patients are summarized in Table 2.

For the 36 patients, a total number of 391 voriconazole *C*_trough_ measurements, with a documented dose of the last voriconazole administration, were available. Of these, 15 measurements were below the LLOQ which was 0.1 mg/L. All children were initially treated with the clinically recommended empirical maintenance dose, followed by individual dose adjustments according to the TDM results:

- 2 to 12 years or 12 to 14 years and <50 kg: 8 mg/kg (7.5 ± 1.6 mg/kg) q12h;
- 12 to 14 years and ≥50 kg or ≥15 years: 4 mg/kg (3.6 ± 1.1 mg/kg) q12h.

**Figure 1:**
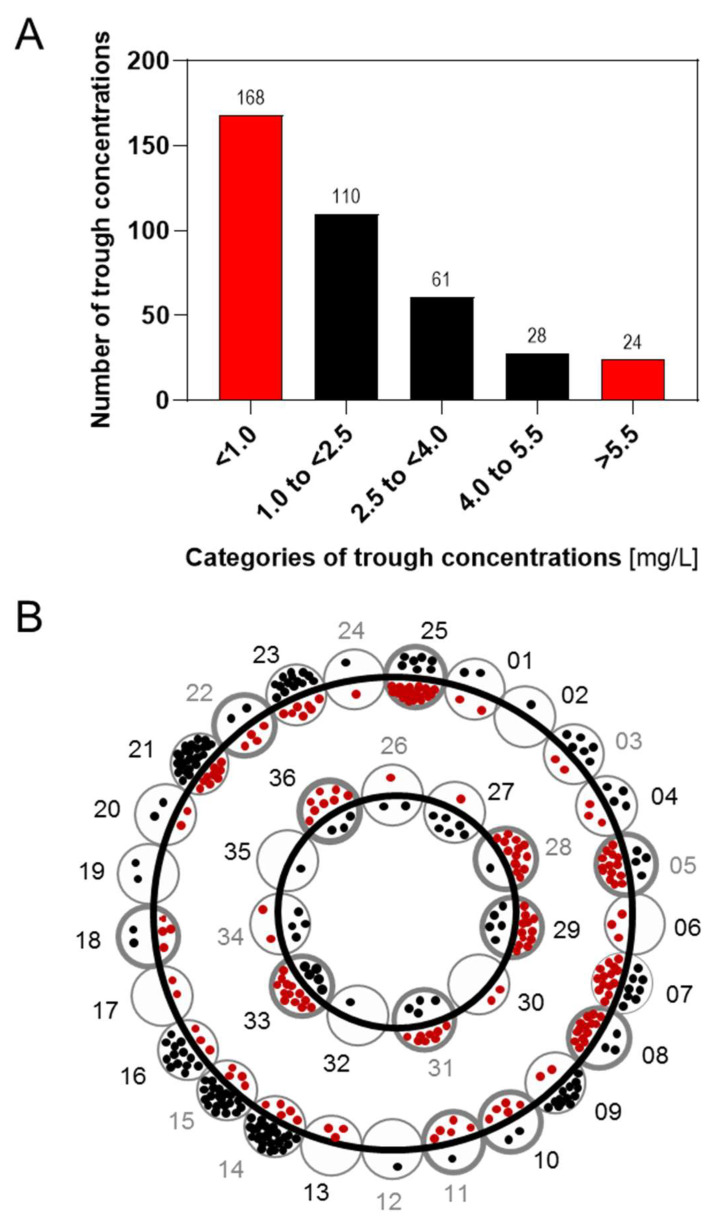
Descriptive analysis of *C*_trough_ determined in children treated with voriconazole. (**A**) Results of therapeutic drug monitoring of voriconazole. (**B**) All voriconazole *C*_trough_ obtained in one individual were rated for the data point being within the range of 1–5.5 mg/L (black dots, within range; red dots, outside range). Individuals without DNA available are labelled in grey. Individuals with >65% of the *C*_trough_ out of range are highlighted by a broader circle line width.

Safe and effective dosing for children younger than 2 years of age has not yet been established, therefore voriconazole was prescribed off-label in 4 patients in this age group. Of the 391 measured voriconazole *C*_trough_, 192 (49.1%) were outside the recommended target *C*_trough_ range of 1–5.5 mg/L, of which 168 (87.5%) were at sub-therapeutic level, and 24 (12.5%) at supra-therapeutic level (Figure 1A). The median number of measurements per patient was 7 (range: 1 to 38). Of the 22 individuals with 6 or more available *C*_trough_ measurements, 45.6% of the *C*_trough_ measurements were out of the range. In 12 of these 22 individuals, >65% of the measured *C*_trough_ were out of the range (Figure 1B).

**Figure 2:**
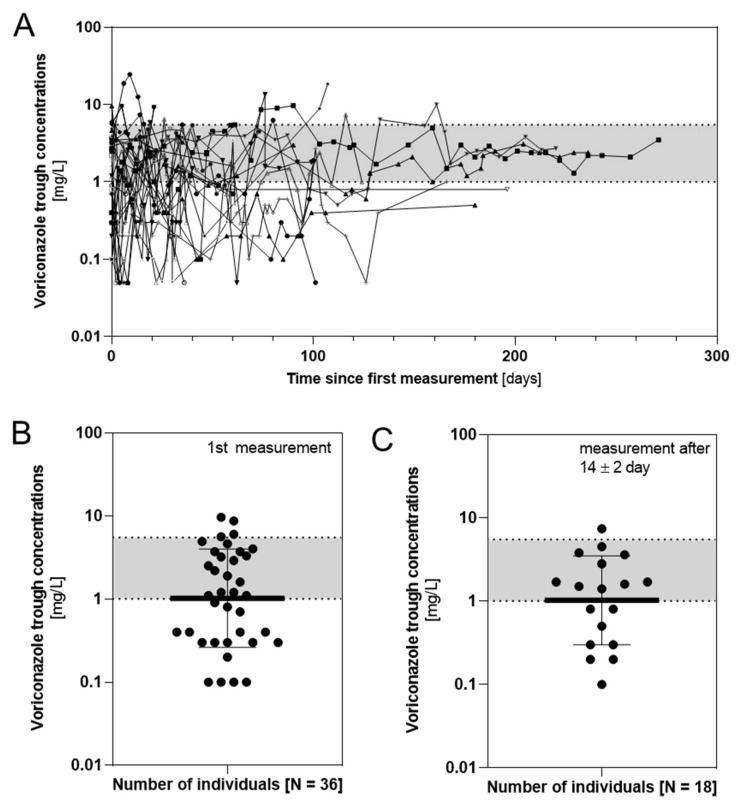
(**A**) *C*_trough_ from therapeutic drug monitoring over time of all 36 individuals. (**B**) First measurement and (**C**) one measurement per patient after 14 ± 2 days of voriconazole *C*_trough_ (*n* = 383; 8 *C*_trough_ were excluded, because they were not measured within the same therapeutic intervention period). On the logarithmic scale of the y-axis, the therapeutic window of *C*_trough_ between 1 and 5.5 mg/L is highlighted in grey.

We are showing the voriconazole *C*_trough_ measured for all 36 patients during TDM in Figure 2A. Values below the LLOQ of 0.1 mg/L were replaced by 0.05 mg/L. As shown in Figure 2B, the *C*_trough_ of the first measurement in all 36 individuals ranged from 0.1 to 9.6 mg/L with a mean ± SD of 2.189 ± 2.429 mg/L. In half of the individuals, a measurement was conducted 14 ± 2 days after the start of the therapeutic drug monitoring. Here, the observed *C*_trough_ ranged from 0.1 to 7.4 mg/L, resulting in a mean ± SD of 1.844 ± 1.931 mg/L (Figure 2C).

**Figure 3:**
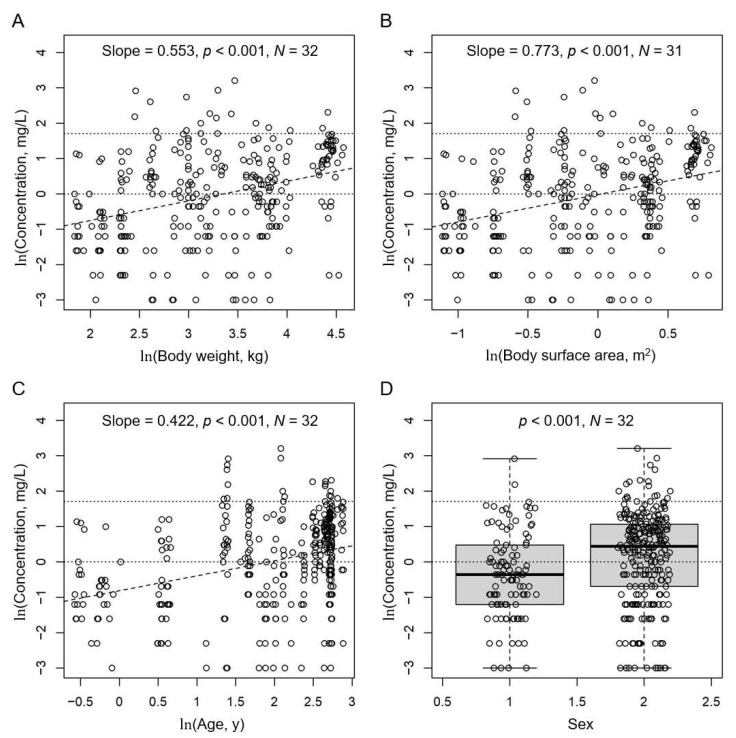
ln(*C*_trough_) correlated with (**A**) ln(body weight), (**B**) ln(body surface area), (**C**) ln(age), and, (**D**) differed significantly between female (f) and male (m) patients. The linear regressions of the correlations in (**A**–**C**) are indicated as broken line and slope with its level of *p*. *p* of a two-tailed homoscedastic t-test in (**D**). Horizontal dotted lines indicate the recommended range for *C*_trough_ (1 to 5.5 mg/L). *N*, numbers of patients with available data and included in the plot.

Plotting ln(*C*_trough_) versus ln(body weight), ln(body surface area) or ln(age) revealed significant positive correlations (Figure 3), indicating that with the current dosing scheme, lighter/younger children have *C*_trough_ below the recommended range more often than children with higher body weight or age, despite the higher recommended dose per body weight at the younger age and lower body weight. The significant difference between ln(*C*_trough_) of female and male patients could be related to this observation (Figure 3D); the mean body weight was 20.8 ± 15.9 kg for female patients and 39.3 ± 24.9 kg for male patients.

### 3.2. Genetic Analysis

For 23 of the 36 pediatric patients from the University Children’s Hospital Zurich, we were able to gather DNA samples retrospectively. The individuals were genotyped for genetic variants within drug metabolizing enzymes and drug transporters as previously suggested [29,30,32]. Analysis of the frequencies in the herein reported study population did not reveal statistically significant differences in the distribution of genotypes or MAF compared to those reported on a European population in the Allele Frequency Aggregator (ALFA) [36]. Only for the SNP rs2331142 (*ABCG2*) there was a tendency for a deviation in frequency (Χ^2^ = 0.089) (Table 3).

### 3.3. Covariate Analysis

We further analyzed the data of the 23 patients with information on genetic polymorphisms by linear mixed effects modelling to account for all potential factors influencing *C*_trough_. Figure S1 shows the distribution of the demographic characteristics of the studied sub-population. The most simple mixed effects model with a linear relationship between ln(*C*_trough_) and ln(administered dose per body weight) revealed the relationship *C*_trough_ = 0.278 × (dose/weight)^0.57^, with the exponent <1 indicating that the ratio between *C*_trough_ and the dose in mg per kg body weight is not constant. This is in agreement with the age- and body-weight dependent dosing scheme. Including body surface area or body weight as a covariate in the model significantly improved the model (*p* < 0.05 for the covariate and −2 LL reduction by >3.84) with effects of 0.8 and 1.2, respectively, and in agreement with Figure 3. Body surface area revealed the lower *p* and −2 LL values in the final model and was thus included as a covariate. The addition of any further demographic parameter (body height, age, sex) or of the factors such as diagnosis or route of administration did not further improve the model.

Figures S2–S4 show the residues plotted against the demographic parameters, co-medications and genetic variants, respectively, after including dose per body weight and body surface area as covariates in the model. Including the covariate body surface area, the putative effect of the sex on ln(*C*_trough_) seen in Figure 3D disappeared (Figure S2), in agreement with a trend towards higher body surface area in male (1.20 ± 0.54 m^2^) than in female (0.77 ± 0.41 m^2^; *p* 0.065) patients. The effects of several co-medicated drugs were tested, in particular if residues differed with *p* < 0.05 and if 3 or more patients received the co-medication. The effects of ciprofloxacin (5 patients/12 *C*_trough_ measurements), levetiracetam (3/27) and propranolol (4/34) remained significant in the final model. All three drugs reduced *C*_trough_ (Table 4). Plotting the remaining residues against the genotypes suggested effects on *C*_trough_ by several of the polymorphisms (Figure S5). Effects were significant for the variant genotypes of ABCC2 rs2273697 (reducing ln(*C*_trough_)), ABCC2 rs717620 (enhancing), ABCG2 rs2231142 (enhancing), CYP2C19 rs4244285 (enhancing), CYP2C19 rs4986893 (enhancing) and CYP3A4 rs35599367 (enhancing). The remaining residues after introducing these co-medications and genes in the model are shown in Figures S5 and S6.

Residues for the individual patient with the polymorphism CYP2C19 rs4244285 AA were in general lower if voriconazole was administered on the same day with metamizole than without this co-medication (Figure S7). In the absence of metamizole co-medication, *C*_trough_ of the AA genotype were in the range of those of the heterozygous carriers. Introducing the interaction term metamizole × CYP2C19 rs4244285 in the model further improved the model with statistical significance. The residues of the final model are plotted against genotypes in Figure S8. Regarding the effects of the genetic variants, it should be noted that only one patient each carried a variant allele for CYP2C19 rs4986893 and CYP3A4 rs35599367 and that the metamizole × CYP2C19 rs4244285 interaction was introduced in the model based on *C*_trough_ measurements with and without metamizole co-medication of one individual patient (carrier of the AA polymorphism).

### 3.4. Final Model

**Figure 4:**
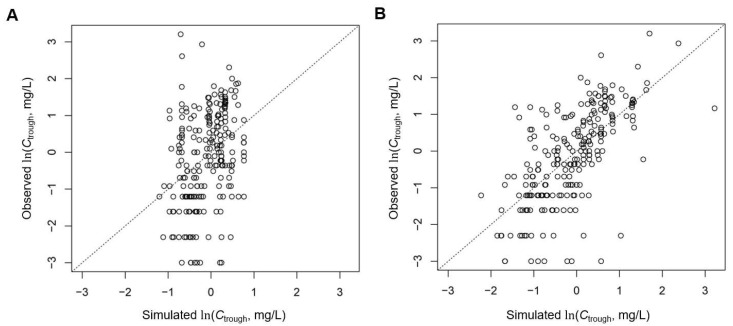
Observed ln(*C*_through_) versus simulated ln(*C*_through_). (**A**) Before inclusion of covariates for co-medications and genetic variants in the model. (**B**) Final model in Table 4. Both plots, population level (no random effects included).

**Figure 5:**
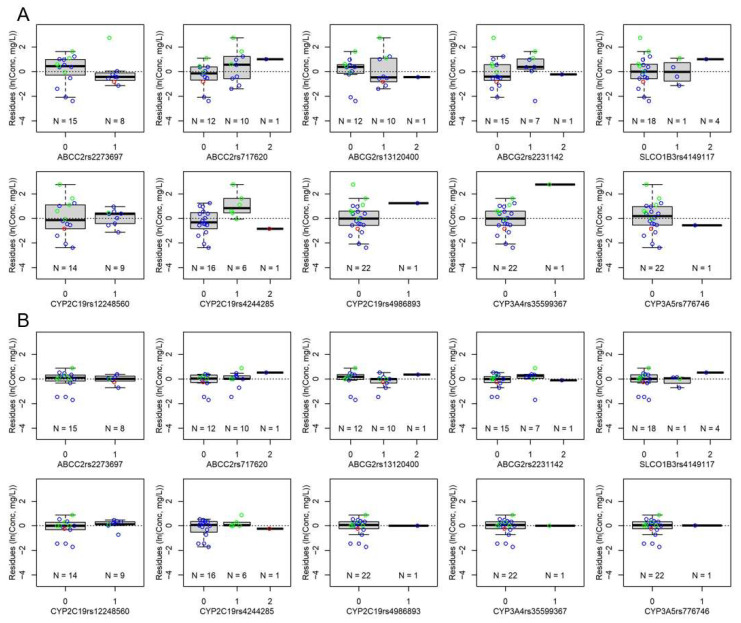
Distribution of the residues of ln(*C*_through_) (observed − predicted) according to the genotypes of the individual patients. (**A**) Before inclusion of covariates for co-medications and genetic variants in the model. (**B**) Final model in Table 4. Both plots, population level (no random effects included). Box-and-whisker plots. Individual data points are superimposed in color. Blue, CYP2C19 rs4244285 GG genotype; green, GA; red, AA. 0, homozygous reference alleles; 1, heterozygous; 2, homozygous variant alleles.

The final model is described in Equation (1) and Table 4. The observed *C*_trough_ are plotted against the simulated *C*_trough_ (without random effects) in Figure 4, in comparison with the model including dose/weight and body surface area only. Figure 5 shows the residues by genotype averaged per patient, before and after including co-medicated drugs and genotypes in the model. Residues with the final model plotted versus the demographic parameters and co-medications are shown in Figures S9 and S10.

log(*C*_trough_) = *θ*_1_ + *θ*_2_ × (ln(dose/weight) − ln(1 mg/kg)) + *θ*_3_ × (ln(surface area) − n(1 m^2^)) + *θ*_4_ × ciprofloxacin + *θ*_5_ × levetiracetam + *θ*_6_ × propranolol + *θ*_7_ × metamizole + *θ*_8_ × ABCC2 rs2273697 + *θ*_9_ × ABCC2 rs717620 + *θ*_10_ × ABCG2 rs2231142 + *θ*_11_ × CYP2C19 rs4244285 + *θ*_12_ × CYP2C19 rs4986893 + *θ*_13_ × CYP3A4 rs35599367 + *θ*_14_ × metamizole × CYP2C19 rs4244285 + (1|ID) (1)

To further corroborate the final model, we excluded the two individual patients with the CYP2C19 rs4986893 and CYP3A4 rs35599367 variant gene, respectively. The remaining effects did not substantially change from those reported in Table 4, indicating robustness of the model (Table S1). While the random effects for the ID intercept were eliminated due to (tolerated) over-parametrization in the final model (Table 4), their distribution was fit to SD 0.342 in the model omitting the two variants with only one patient each (Table S1). The *p* values of the effects of propranolol, ABCC2 s717620 and the metamizole × CYP2C19 rs4244285 interaction, however, increased to between 0.05 and 0.1. Furthermore, Table S2 shows the results without the metamizole × CYP2C19 rs4244285 interaction effect. The difference was highest for the effect of CYP2C19 rs4244285, which was by 0.319 lower without the interaction term. Differences for all other effects were within ± 0.13. We furthermore compared the final model with a model without effects for co-medication (Table S3). The effects of the genetic polymorphisms were confirmed. As expected, they changed in effect size and *p* for the effects of CYP2C19 rs4244285 and ABCC2 rs2273697 increased to between 0.05 and 0.1.

## 4. Discussion

In this study we analyzed the routine TDM data of 36 pediatric patients treated with voriconazole. A relevant proportion of the TDM data were out of the recommended range and this proportion remained at a comparable level over the therapy duration. Although the therapeutic window is defined, the fact that voriconazole pharmacokinetics are highly variable makes it difficult to define the appropriate dose during therapy. This high variability could also be seen in our data at the beginning and still after two weeks of treatment under TDM.

The recommended dose per body weight is higher in younger children and children with low body weight. The occurrence of sub-therapeutic *C*_trough_ increased with decreasing body weight in our study, when plotting ln(*C*_trough_) versus ln(body weight). A similar effect was found in the final mixed effects model which suggests that *C*_trough_ increases with (body surface area/1 m^2^)^1.14^. The exponent 1.14 indicates near proportionality between *C*_trough_ and body surface area, despite the age- and body-weight adjusted dosing scheme. Dosing schemes may thus require further adjustments, in particular for the lower age and body weight ranges. The predicted *C*_trough_ at the reference parameters in the final mixed effects model (body surface area, 1 m^2^; no influencing co-medication or polymorphism, dose per body weight, 8 mg for age <12 y) was 0.80 mg/L (exp(*θ*_1_) × (8 mg)^*θ*_2_^). It was similar (0.97 mg/L) in the model with dose per body weight and surface area as sole effects. This is in agreement with the ln(*C*_trough_) versus ln(body surface area) plot of 31 patients and is close to the recommended lower limit of therapeutic *C*_trough_. However, the highly significant effect of body surface area in the final mixed-effects model confirmed the finding from the simple correlation: at the current dosing recommendation, predicted *C*_trough_ are considerably lower than 1 mg/L at body surface area <1 m^2^.

Both, the simple correlation and the mixed effects model with dose per body weight and body surface area as sole effects were not able to explain the high inter-individual and for some patients intraindividual variability in *C*_trough_. Including the route of administration or the secondary diagnose in the model did not improve the predictions.

In this retrospective data analysis, we focused on the influence of SNPs and co-medications in addition to the available patient-specific characteristics. We selected SNPs within CYPC19 and CYP3A4 for which an association with voriconazole blood levels has been shown by other groups [29,30,37] and added SNPs of CYP3A5 as its role in the voriconazole pharmacokinetics is uncertain and further studies were requested [38].

The metabolizing enzyme CYP2C19 is the key enzyme catalyzing the formation of the inactive voriconazole N-oxide [3]. CYP2C19 is polymorph and genetic variants are known to impact voriconazole’s pharmacokinetics, so that the Clinical Pharmacogenetics Implementation Consortium (CPIC) published dosing recommendations for voriconazole treatment based on the CYP2C19 genotype for pediatric patients summarizing the evidence from the literature [27,28,32,39]. We genotyped 4 different SNPs of this enzyme. None of the patients harboured the polymorphism CYP2C19 rs28399504 (c.1A > G). Of the remaining 3 SNPs in the CYP2C19 gene, rs4244285 and rs4986893 were identified as covariates in our final model, with a significant influence on voriconazole *C*_trough_.

CYP2C19 rs4244285 (c.681G > A) is a splicing defect variant, leads to a poor metabolizer phenotype if an individual carries two no function alleles and consequently increases trough levels [40]. Our model suggested that this polymorphism in general increased *C*_trough_. However, the only patient who was homozygous carrying two no function alleles, had a lower median C_trough_ than the median *C*_trough_ of the 6 heterozygous patients and even of the non-carriers. The *C*_trough_ of the rs4244285 AA carrier showed a surprisingly high intraindividual variability, though. This inspired us to search for potential interaction effects for this SNP in the model and identified metamizole co-medication as potential confounder. While it had no significant effect on *C*_trough_ in general, its interaction with CYP2C19 rs4244285 was significant in the final model. In adult patients who are lacking CYP2C19, an increased role for CYP3A4 in the N-oxidation of voriconazole is suggested [41]. Metamizole is a weak to moderate inducer of several CYP isozymes, including CYP3A4, CYP2C19 and CYP2C9 [42]. Based on our findings, we speculate that the induction of CYP3A4 (and possibly other CYP isozymes) became relevant in the patient homozygous for the rs4244285 AA polymorphism when co-medicated with metamizole. This would explain the clustering towards higher *C*_trough_ in the absence, and lower *C*_trough_ in the presence of the metamizole co-medication in this individual patient. However, our hypothesis requires confirmation as it is based on one individual patient. Omission of this interaction in the final model had no major impact on the sizes of the remaining effects. Residues were similar for metamizole co-medicated and metamizole-naive heterozygous carriers of the rs4244285 polymorphism. According to the recent study [43], metamizole metabolism itself depends on the CYP2C19 genotype. We dare to speculate here that metamizole concentrations were only high enough in the homozygous CYP2C19 rs4244285 AA carrier to substantially induce CYP3A4 expression.

CYP2C19 rs4986893 (c.636G > A) is a loss-of-function mutation with predicted higher substrate concentrations as well [40]. Although we had only one patient in our population heterozygous for this variant, we considered it in the final model. The resulting *C*_trough_-increasing effect was in agreement with the expectation. Omitting this polymorphism had no major impact on the final model. CYP2C19 rs12248560 g.-806C > T is associated with accelerated metabolism [40], which we could not confirm in any of our tested models, including the final model. Espinoza et al. came to a contrary conclusion in a study with immunocompromised children [44]. In their study, the averaged *C*_trough_ was lower in carriers than non-carriers. However, as the authors discussed themselves, they did not exclude potential carriers of reduced-function CYP2C19 polymorphisms from the control group for the comparison, leaving the question unanswered whether the difference was due to the rs12248560 genotype or due to other polymorphisms in the control group (in addition). Whether the rs12248560 mutation in the promotor region results in increased CYP2C19 expression in children as observed in adults remains to be shown [45]. Our data from 9 heterozygous carriers (compared to 14 non-carriers) would not suggest that.

The CYP3A4 rs35599367 C > T in intron 6 occurs with a frequency of 5–7% in the Caucasian population and carriers are assumed to have lower hepatic CYP3A4 expression and activity [46]. In our population, only one patient carried the CT variant. This child had also a loss of function mutation in CYP2C19 rs4244285 (GA genotype). Extremely high *C*_trough_ (up to 24.6 mg/L) were measured, even after reducing the dose and the therapy had to be discontinued due to neurotoxicity. We included the polymorphism in our final model although it was represented by only one patient. Excluding it from the final model had no major impact on the remaining effects. No influence on voriconazole *C*_trough_ was found for the genetic variant CYP3A5 rs776746 (g.6986A > G) and none of the patients harboured the variant CYP3A5 rs10264272 (g.14690G > A).

In addition to the voriconazole metabolizing enzymes our selection of genetic variants included genes encoding for drug transporters *ABCC2*, *ABCG2*, and *SLCO1B3* with a known or suspected role in voriconazole pharmacokinetics [30,47].

The ATP binding cassette (ABC) transporter multidrug resistance-associated protein 2 (MRP2) encoded by the *ABCC2* gene is expressed on the apical membranes of the intestinal epithelia, the kidney proximal tubules, and the canalicular membrane of hepatocytes, where it governs biliary excretion of its substrates [48]. In vitro data do not support that voriconazole interacts with MRP2 [49]. However, Zeng et al. observed an influence of the genetic variant ABCC2 rs2273697 (c.1249G > A), that increases the activity of the transporter [50], on voriconazole concentrations in patients undergoing hematopoietic stem cell transplantation [47]. We confirmed this effect in our study which included 8 heterozygous pediatric patients; the ABCC2 rs2273697 polymorphism had a significant negative effect on *C*_trough_ in the final model. The influence of MRP2 on voriconazole pharmacokinetics is further substantiated by our study as we also confirmed that the variant ABCC2 rs717620 (c.-24C > T), which is associated with lower expression levels of MRP2 [51], has an *C*_trough_ increasing effect as previously described by Allegra et al. directly comparing carriers and non-carriers in a Student’s t-test [30]. The rs717620 polymorphism is also known for its association with methotrexate toxicity in childhood acute lymphoblastic leukemia [52]. Our results for the two SNPs rs2273697 and rs717620 in the *ABCC2* gene support its influence on voriconazole *C*_trough_. However, it remains to be tested whether voriconazole is indeed a substrate of the MRP2 transporter in vivo.

The breast cancer resistance protein (BCRP) is a drug transporter encoded by the *ABCG2* gene and is expressed in the sinusoidal membrane of hepatocytes where it affects plasma clearance through hepatobiliary elimination, and in the apical membrane of enterocytes where it limits oral bioavailability through intestinal elimination [53]. Overexpression of BCRP in cancer cells is associated with high levels of resistance to various anticancer drugs [54]. There are no in vitro data supporting interaction of voriconazole with BCRP [49,55]. Nevertheless, we included two different genetic variants of ABCG2 in the genetic testing, as Allegra et al. had reported that ABCG2 rs13120400 (c.1194 + 928T > C), although it does not belong to the major ABCG2 genetic variants of known clinical relevance [56], had an influence on voriconazole *C*_trough_ and values were increased in children carrying the ABCG2 rs13120400 CC genotype. While the rs13120400 polymorphism had no significant effect in our study, the ABCG2 rs2231142 (c.421C > A) missense mutation, which is suggested to reduce level and function of BCRP [56], significantly increased voriconazole *C*_trough_ in 7 heterozygous and 1 homozygous patient. Our study adds evidence that besides MRP2 (*ABCC2)* also BCRP (*ABCG2)* is involved in voriconazole pharmacokinetics.

In addition to the members of the ABC-transporter family, our selection also included a genetic variant located within the gene encoding for the hepatic uptake transporter OATP1B3 [57]. Allegra et al. reported that the genetic variant SLCO1B3 rs4149117 (c.334G > T) is associated with significantly reduced *C*_trough_ values in 4 carriers either hetero- or homozygous for the variant allele. Our study including 4 heterozygous and 1 homozygous carriers did not confirm this effect.

While we found ample agreement between our study and published results regarding the effects of genetic polymorphisms, the identified effects of co-mediations in our study were entirely unexpected. Ciprofloxacin, levetiracetam and propranolol had each a significant reducing effect on voriconazole *C*_trough_. The strength of the effects was in the range of that of the genetic polymorphisms. The three drugs have a relatively high dose in common, favoring drug-drug interactions of any kind. Possible mechanisms involved in drug-related concentration reduction are (i) the induction of enzyme or transporter expression, (ii) the increase in blood flow in the eliminating organ and (iii) the competition for plasma-protein binding. All three mechanisms may increase a co-medicated drug’s clearance and point iii may result in lower serum concentrations due to an increase in volume of distribution [26]. Voriconazole is a low-extraction drug (clearance << organ plasma flow) [24]; an increase in organ blood flow would, therefore, not affect its pharmacokinetics sustantially, excluding this as a probable mechanism. Ciprofloxacin is an inhibitor of CYP isozymes [58], but not known as an enzyme or transporter inducer and plasma protein binding is considered irrelevant regarding drug-drug interactions [59]. For levetiracetam, the available in vitro reports on its CYP-inducing effects are controversial [60,61]. Nevertheless, in clinical use, levetiracetam is considered to be a non-enzyme-inducing antiepileptic drug for which CYP enzymes play a minor role in elimination [62,63,64]. However, its pharmacokinetics appear to be influenced by the co-administration of CYP-inducers (enzyme inducing AEDs) [65]; the underlying mechanisms require further investigation. Propranolol is a substrate of several CYP isozymes [58], but not known as an inducer of CYPs or transporter proteins. Drug-drug interactions with propranolol based on competitive plasma protein binding are not known. We searched our data for potential effects of combinations of ciprofloxacin, levetiracetam or propranolol with other drugs, but did not find any. Searching the literature did not reveal any known drug-drug interactions between voriconazole and the three drugs. Factors related to these co-medications, such as their indications, may have resulted in their significant effects in the mixed-effects model. However, the indications for these drugs were not always available from the extracted data.

All significant effects in the model were above 0.41 (below −0.58, respectively). This corresponds to increases in *C*_trough_ of 50% or more (>exp(0.41)) and reductions by 44% or more. We, therefore, consider all identified effects as potentially clinically relevant. The *C*_trough_-increasing effects were highest for body surface area (proportional) and CYP2C19 rs4244285 polymorphism (*C*_trough_ 2.5-fold higher). The *C*_trough_-reducing effects were strongest for levetiracetam and ciprofloxacin co-medications, both reducing *C*_trough_ to 35–40% of controls, followed by the ABCC2 rs2273697 polymorphism, reducing *C*_trough_ to ~50% of the controls. These estimates are for the heterozygous carriers. The effects of the polymorphisms with only one carrier were not included in this ranking. Based on these findings, we recommend adjusting the current dosing scheme to achieve higher *C*_trough_ in children with body surface area <1 m^2^. However, a prospective study needs to show whether this is better achieved by increasing the dose or by reducing the dosing interval. The first approach may result in toxic peak levels, the latter in drug accumulation. The right choice can only be made based on the voriconazole pharmacokinetics in this sub-group of pediatric patients, i.e., based on more concentration measurements between drug administrations. We furthermore recommend to genotype for CYP2C19 rs4244285 and potentially ABCC2 rs2273697. As both polymorphisms affect the elimination, adjusting the dosing interval may be more sensible than adjusting the dose. The CYP2C19 rs4244285 polymorphism would require prolongation, the ABCC2 rs2273697 polymorphism reduction of the dosing interval. As the strong effects of the co-medicated ciprofloxacin, levetiracetam and propranolol where unexpected, we highly suggest to closely monitor voriconazole *C*_trough_ in combination with these drugs and adjust dosing schemes accordingly.

Our study was retrospective, and a limited number of patients were included. Some of the identified effects were based on <3 patients. To exclude any bias from these effects, the final model was confirmed after excluding these effects/patients. The high number of co-medicated drugs in these patients rendered the analysis of effects from co-medicated drugs challenging. The unexpected findings for co-medications in our model need thus to be interpreted with care.

## 5. Conclusions

Our results suggest that children with body surface area <1 m^2^ are at highest risk for *C*_trough_ below the recommended 1 mg/L. Our study confirmed the well accepted effect of the CYP2C19 genotype on voriconazole serum concentrations. It furthermore suggests effects on the concentrations by polymorphisms in the ABCC2 and ABCG2 transporters. Further effects by polymorphic CYP3A4 and co-medication with ciprofloxacin, levetiracetam and propranolol, as well as a CYP2C19-genotype dependent metamizole effect need to be confirmed. Our study provides evidence that the high inter- and intraindividual variability in voriconazole serum concentrations are predictable and that, therefore, prospective pharmacokinetics studies will allow the refinement of the dosing recommendations for seriously ill children. For the prescription of an optimal drug dosing scheme, in addition to therapeutic drug monitoring pre-emptive PGx testing for CYP2C19 is recommended and potentially genotyping for ABCC2 could in the future play a significant role in clinical routine. Attention should also be paid to a careful selection of co-medications in patients suffering from IFI and treated with voriconazole.

## Figures and Tables

**Table 1:** Summary of genetic variants determined applying commercially available TaqMan^®^ assays (primer/probe-mixes) in this study.

SNP-Identifier	Gene	Genetic Variant	Assay ID	*
rs2273697	*ABCC2*	c.1249G > A	C__22272980_20	
rs717620	*ABCC2*	c.24C > T	C___2814642_10	
rs13120400	*ABCG2*	c.1194 + 928T > C	C___9510480_10 ^a^	
rs2231142	*ABCG2*	c.421C > A	C__15854163_70	*
rs12248560	*CYP2C19*	* 17; g.-806C > T	C___469857_10	
rs28399504	*CYP2C19*	* 4; c.1A > G	C__30634136_10	
rs4244285	*CYP2C19*	* 2; c.681G > A	C__25986767_70	
rs4986893	*CYP2C19*	* 3; c.636G > A	C__27861809_10	
rs35599367	*CYP3A4*	* 22; (intronic) C > T	C__59013445_10 ^a^	*
rs10264272	*CYP3A5*	* 6; g.14690G > A	C__30203950_10	*
rs776746	*CYP3A5*	* 3; g.6986A > G	C__26201809_30	*
rs4149117	*SLCO1B3*	c.334G > T	C__25639181_40	

Assays tagged with a * are located on the reverse strang; results were translated into coding strang prior to submitting the data to the *SwissPK*^cdw^ tenant at Leonhard Med. ^a^, indicates primer/probe-mix delivered at 40× concentration.

**Table 2:** Characteristics of the patient population at the beginning of voriconazole therapy.

Female	30.6	%	*N* = 11
Male	69.4	%	*N* = 25
Age, median (range)	10 (0–17)	years	*N* = 36
Age, mean ± SD	9.6 ± 5.4	years	*N* = 36
Body weight, median (range)	30.6 (6.5–96.9)	kg	*N* = 36
Body weight, mean ± SD	35.5 ± 21.8	kg	*N* = 36
Body surface area *, (median (range)	1.09 (0.33–2.25)	m^2^	*N* = 35
Body surface area *, mean ± SD	1.13 ± 0.49	m^2^	*N* = 35
Height, median (range)	139.5 (62–188)	cm	*N* = 35
Height, mean ± SD	133.2 ± 34.0	cm	*N* = 35
Voriconazole i.v.			*N* = 21
Voriconazole p.o.			*N* = 15
Confirmed invasive aspergillosis (IA)			*N* = 23
Probable or suspected IA			*N* = 7
Prophylaxis of IA			*N* = 3
Other IFI			*N* = 3

* Body surface area was calculated using the Mosteller’s equation [35]. *N*, number of individuals.

**Table 3:** Summary of the genetic analysis reporting the number of individuals and the number of *C*_trough_ measurements observed for each genotype (*n* = 251; 11 *C*_trough_ values were excluded because of lacking information on the dose or because an additional dose was administered >2 h after the initial dose within a dosing interval). Reported is the observed MAF, and the results of the Χ^2^-test.

SNP-Identifier	Gene	Genotype: Number of Individuals/*C*_trough_ Measurements			MAF Observed	MAF Reported *	*Χ* ^2 a^	*Χ* ^2 b^
rs2273697	*ABCC2*	GG: 15/135	GA: 8/105	AA: 0	0.1739	0.2014	0.601	0.610
rs717620	*ABCC2*	CC: 12/143	CT: 10/96	TT: 1/1	0.2609	0.1997	0.830	0.480
rs13120400	*ABCG2*	TT: 12/140	TC: 10/83	CC: 1/17	0.2609	0.2185	0.830	0.809
rs2231142	*ABCG2*	CC: 15/161	CA: 7/47	AA: 1/32	0.1957	0.1026	0.988	0.089
rs12248560	*CYP2C19*	CC: 14/157	CT: 9/83	TT: 0	0.1957	0.2314	0.506	0.515
rs28399504	*CYP2C19*	AA: 23/240	AG: 0	GG: 0	n.d.	0.0033	n.a.	0.927
rs4244285	*CYP2C19*	GG: 16/161	GA: 6/64	AA: 1/15	0.1739	0.1473	0.907	0.762
rs4986893	*CYP2C19*	GG: 22/224	GA: 1/16	AA: 0	0.0217	0.0058	0.994	0.358
rs35599367	*CYP3A4*	CC: 22/237	CT: 1/3	TT: 0	0.0217	0.0462	0.994	0.732
rs10264272	*CYP3A5*	GG: 23/240	GA: 0	AA: 0	n.d.	0.0011	n.a.	0.975
rs776746	*CYP3A5*	GG: 22/216	GA: 1/24	AA: 0	0.0217	0.0700	0.994	0.435
rs4149117	*SLCO1B3*	GG: 18/207	GT: 4/32	TT: 1/1	0.1304	0.1411	0.535	0.563

* ALFA Allele Frequency https://www.ncbi.nlm.nih.gov/snp/docs/gsr/alfa, accessed on 22 March 2021; selected were the frequencies reported for the European population; ^a^, comparing the number of individuals observed and calculated from the observed MAF; ^b^, comparing the number of individuals observed and expected according to the ALFA MAF. n.d., not detected; n.a., not applicable.

**Table 4:** Fit parameters with SE and *p* of the final model.

Parameter	Reference Value for Intercept	Fit Effect	SE	*p*	Significance ^a^
**Fixed Effects**					
*θ*_1_, Intercept (log(*C*_trough_))		−2.0763	0.4637	<10^−4^	***
*θ*_2_, ln(dose/weight, mg/kg)	ln(1 mg/kg)	0.8905	0.2004	<10^−4^	***
*θ*_3_, Δ ln(surface area, m^2^)	ln(1 m^2^)	1.1437	0.2388	<10^−5^	***
*θ*_4_, ciprofloxacin	No ciprofloxacin	−0.9497	0.2925	0.0013	**
*θ*_5_, levetiracetam	No levetiracetam	−1.0043	0.2850	0.00051	***
*θ*_6_, propranolol	No propranolol	−0.5887	0.2671	0.028	*
*θ*_7_, metamizole	No metamizole	−0.1520	0.1673	0.36	
*θ*_8_, ABCC2 rs2273697	ABCC2 rs2273697 GG	−0.6581	0.1708	0.00015	***
*θ*_9_, ABCC2 rs717620	ABCC2 rs717620 CC	0.4130	0.1918	0.032	*
*θ*_10_, ABCG2 rs2231142	ABCG2 rs2231142 CC	0.4481	0.1391	0.0015	**
*θ*_11_, CYP2C19 rs4244285	CYP2C19 rs4244285 GG	0.8990	0.1980	<10^−5^	***
*θ*_12_, CYP2C19 rs4986893	CYP2C19 rs4986893 GG	1.6289	0.4302	0.00019	***
*θ*_13_, CYP3A4 rs35599367	CYP3A4 rs35599367 CC	3.3737	0.6203	<10^−6^	***
*θ*_14_, metamizole × CYP2C19 rs4244285	No metamizole, CYP2C19 rs4244285 GG	−0.4911	0.2231	0.029	*
**Random effects distribution (SD) and Residuals**					
ID intercept, n.a.; Residuals, 0.9385					

^a^ *, *p* < 0.05; **, *p* < 0.005; ***, *p* < 0.0005; n.a., not applicable (see text).

## Data Availability

Secure access to the data presented in this study is available on request according to the data sharing policies of SPHN. The data are not publicly available due to confidentiality reasons.

## Supplementary Materials

The following supporting information can be downloaded at: https://www.mdpi.com/article/10.3390/pharmaceutics14061289/s1, Figure S1: Demographic characteristics of the sub-population with information on genetic polymorphisms, analyzed by linear mixed effects modelling; Figure S2: Residues (observed ln(*C_trough_*) minus simulated ln(*C_trough_*)) plotted against demographic parameters as indicated after including dose per body weight and body surface area as covariates in the model; Figure S3: Residues plotted against co-medication as indicated after including dose per body weight and body surface area as covariates in the model; Figure S4: Residues plotted against gene variants as indicated after including dose per body weight and body surface area as covariates in the model; Figure S5: Residues plotted as in Supplementary Figure S4, but including co-medication with ciprofloxacin, levetiracetam and propranolol as covariates; Figure S6: As Supplementary Figure S5, but in addition including the significant effects of the genetic variants in the model; Figure S7: Residues shown in Figure S6 plotted versus the CYP2C19 rs4244285 genotype; Figure S8: As Supplementary Figure S6, but for the final model, including the interaction term metamizole×CYP2C19 rs4244285 in the model; Figure S9: Residues from the final model plotted versus demographic parameters; Figure S10: Residues from the final model plotted versus co-medication. Colors, as in Supplementary Figure S3; Table S1: Final model after exclusion of the two carriers of a CYP2C19 rs4986893 or CYP3A4 rs35599367 variant gene; Table S2: Final model, but without the metamizole × CYP2C19 rs4244285 interaction; Table S3: Final model, but without effects for co-medication.
