# Supplementary material for: Pharmacogenetic Analysis of Voriconazole Treatment in Children
Source: Pharmaceutics. 2022 Jun 17;14(6):1289. doi: 10.3390/pharmaceutics14061289 (PMC9227859; doi:10.3390/pharmaceutics14061289)
Supplement: Supplementary file 1 [file pharmaceutics-14-01289-s001.zip › pharmaceutics-1764204-supplementary.pdf]

Pharmacogenetic Analysis of Voriconazole Treatment in Children

Romy Tilen [1,2], Paolo Paioni [1], Aljoscha N. Goetschi [3], Roland Goers [2], Isabell Seibert [2], Daniel Müller [4], Julia A. Bielicki [5], Christoph Berger [1], Stefanie D. Krämer [3] and Henriette E. Meyer zu Schwabedissen [2]

[1] Division of Infectious Diseases and Hospital Epidemiology, University Children's Hospital Zurich, Steinwiesstrasse 75, 8032 Zurich, Switzerland; romy.tilen@kispi.uzh.ch (R.T.); paolo.paioni@kispi.uzh.ch (P.P.); christoph.berger@kispi.uzh.ch (C.B.)
[2] Biopharmacy, Department Pharmaceutical Sciences, University Basel, Klingelbergstrasse 50, 4056 Basel, Switzerland; romy.tilen@unibas.ch (R.T.); roland.goers@unibas.ch (R.G.); isabell.seibert@unibas.ch (I.S.); h.meyerzuschwabedissen@unibas.ch (H.E.M.z.S.)
[3] Biopharmacy, Institute of Pharmaceutical Sciences, Department of Chemistry and Applied Biosciences, ETH Zurich, Vladimir-Prelog-Weg 4, 8093 Zurich, Switzerland; goetscha@student.ethz.ch (A.N.G.); stefanie.kraemer@pharma.ethz.ch (S.D.K.)
[4] Institute of Clinical Chemistry, University Hospital Zurich, Rämistr. 100, 8091 Zurich, Switzerland; daniel.mueller@usz.ch (D.M.)
[5] Paediatric Research Centre, University Children's Hospital Basel, Basel, Spitalstrasse 33, 4056 Basel, Switzerland; juliaannabielicki@ukbb.ch (J.A.B.)
* Correspondence: romy.tilen@kispi.ch (R.T.); h.meyerzuschwabedissen@unibas.ch (H.E.M.z.S.)

Supplementary Information

Supplementary Figures

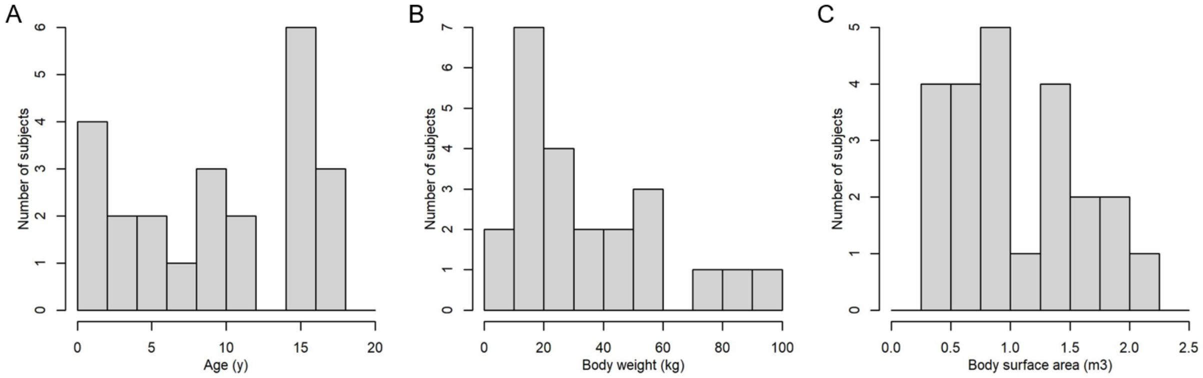

Figure S1. Demographic characteristics of the sub-population with information on genetic polymorphisms, analyzed by linear mixed effects modelling. Distribution for age (**A**), body weight (**B**) and body surface area (**C**).

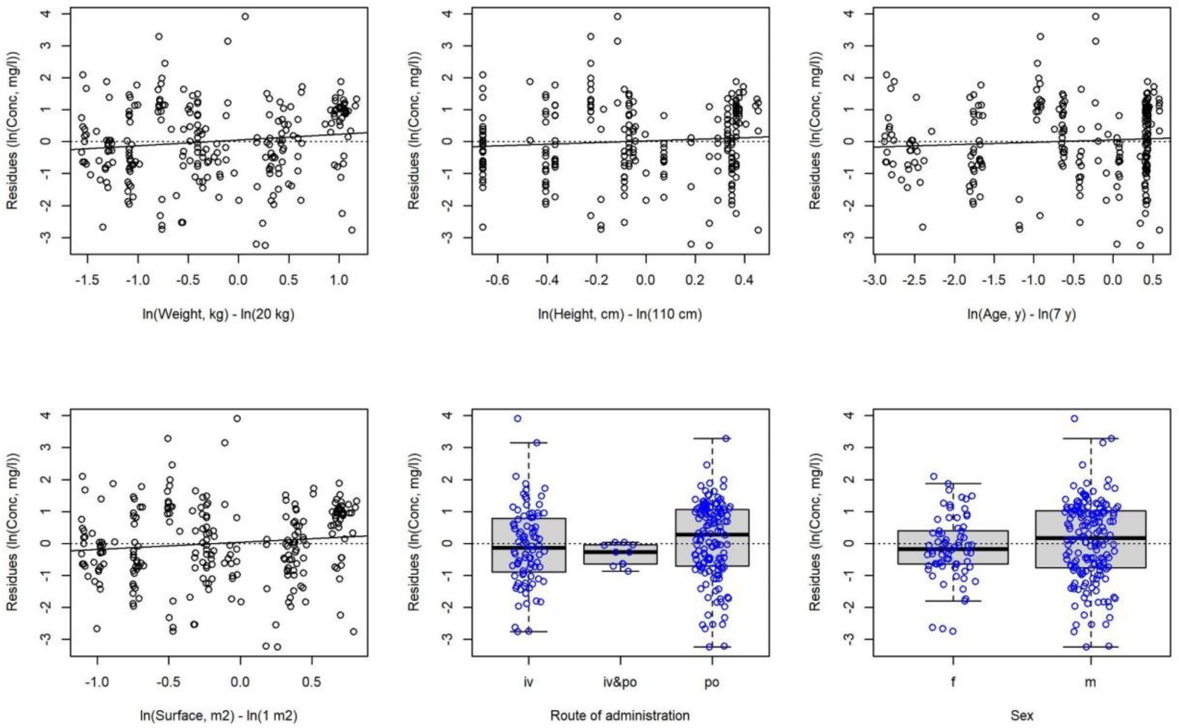

Figure S2. Residues (observed ln(C_{trough}) minus simulated ln(C_{trough})) plotted against demographic parameters as indicated after including dose per body weight and body surface area as covariates in the model.

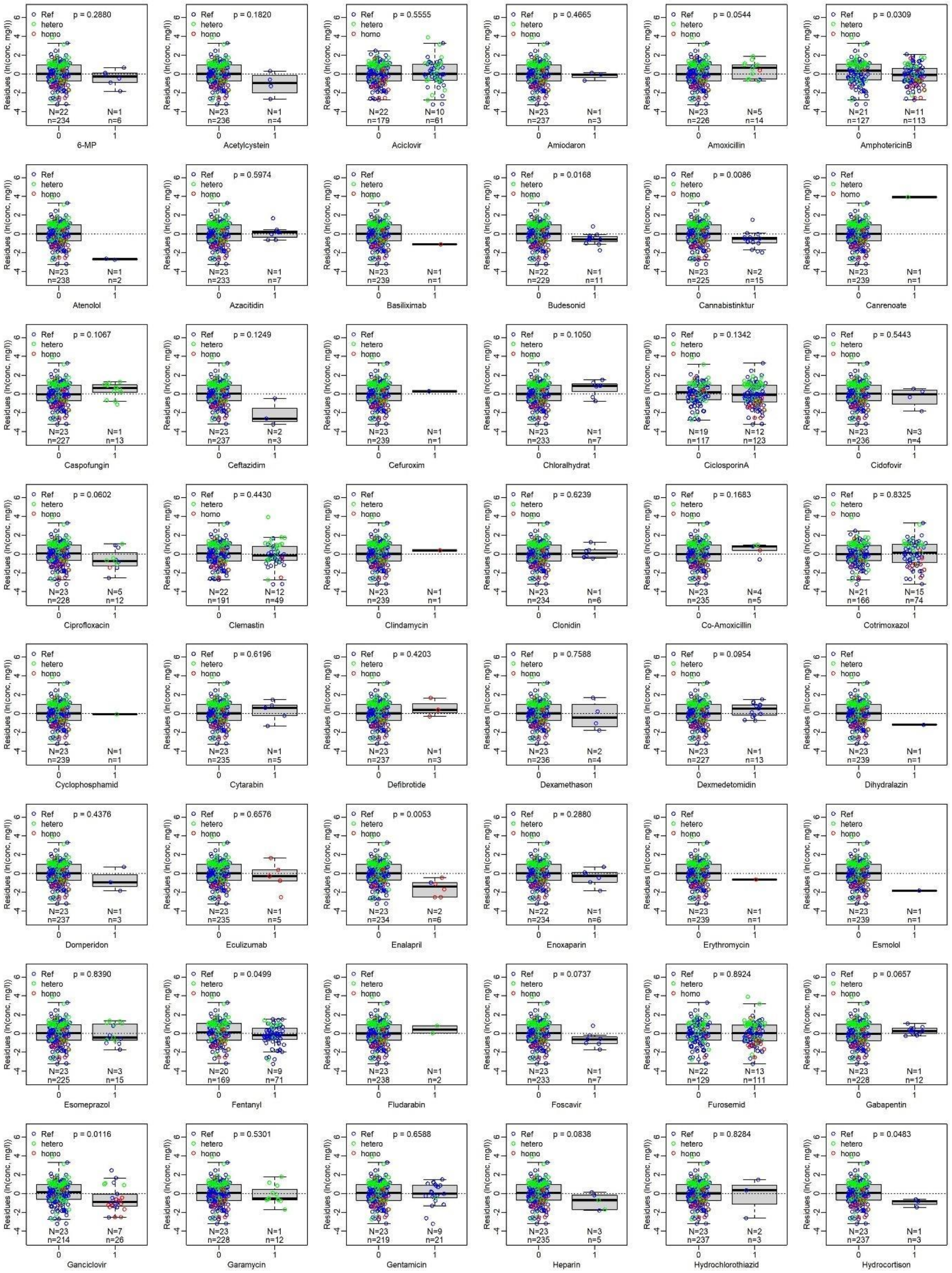
Residues (ln(conc, mg/l))
Ref
hetero
homo
6-MP p = 0.2880 N=22 n=234 N=1 n=6
Acetylcystein p = 0.1820 N=23 n=236 N=1 n=4
Aciclovir p = 0.5555 N=22 n=179 N=10 n=61
Amiodaron p = 0.4665 N=23 n=237 N=1 n=3
Amoxicillin p = 0.0544 N=23 n=226 N=5 n=14
AmphotericinB p = 0.0309 N=21 n=127 N=11 n=113
Atenolol N=23 n=238 N=1 n=2
Azacitidin p = 0.5974 N=23 n=233 N=1 n=7
Basiliximab N=23 n=239 N=1 n=1
Budesonid p = 0.0168 N=22 n=229 N=1 n=11
Cannabistinktur p = 0.0086 N=23 n=225 N=2 n=15
Canrenoate N=23 n=239 N=1 n=1
Caspofungin p = 0.1067 N=23 n=227 N=1 n=13
Ceftazidim p = 0.1249 N=23 n=237 N=2 n=3
Cefuroxim N=23 n=239 N=1 n=1
Chloralhydrat p = 0.1050 N=23 n=233 N=1 n=7
CiclosporinA p = 0.1342 N=19 n=117 N=12 n=123
Cidofovir p = 0.5443 N=23 n=236 N=3 n=4
Ciprofloxacin p = 0.0602 N=23 n=228 N=5 n=12
Clemastin p = 0.4430 N=22 n=191 N=12 n=49
Clindamycin N=23 n=239 N=1 n=1
Clonidin p = 0.6239 N=23 n=234 N=1 n=6
Co-Amoxicillin p = 0.1683 N=23 n=235 N=4 n=5
Cotrimoxazol p = 0.8325 N=21 n=166 N=15 n=74
Cyclophosphamid N=23 n=239 N=1 n=1
Cytarabin p = 0.6196 N=23 n=235 N=1 n=5
Defibrotide p = 0.4203 N=23 n=237 N=1 n=3
Dexamethason p = 0.7588 N=23 n=236 N=2 n=4
Dexmedetomidin p = 0.0954 N=23 n=227 N=1 n=13
Dihydralazin N=23 n=239 N=1 n=1
Domperidon p = 0.4376 N=23 n=237 N=1 n=3
Eculizumab p = 0.6576 N=23 n=235 N=1 n=5
Enalapril p = 0.0053 N=23 n=234 N=2 n=6
Enoxaparin p = 0.2880 N=22 n=234 N=1 n=6
Erythromycin N=23 n=239 N=1 n=1
Esmolol N=23 n=239 N=1 n=1
Esomeprazol p = 0.8390 N=23 n=225 N=3 n=15
Fentanyl p = 0.0499 N=20 n=169 N=9 n=71
Fludarabin N=23 n=238 N=1 n=2
Foscavir p = 0.0737 N=23 n=233 N=1 n=7
Furosemid p = 0.8924 N=22 n=129 N=13 n=111
Gabapentin p = 0.0657 N=23 n=228 N=1 n=12
Ganciclovir p = 0.0116 N=23 n=214 N=7 n=26
Garamycin p = 0.5301 N=23 n=228 N=1 n=12
Gentamicin p = 0.6588 N=23 n=219 N=9 n=21
Heparin p = 0.0838 N=23 n=235 N=3 n=5
Hydrochlorothiazid p = 0.8284 N=23 n=237 N=2 n=3
Hydrocortison p = 0.0483 N=23 n=237 N=1 n=3

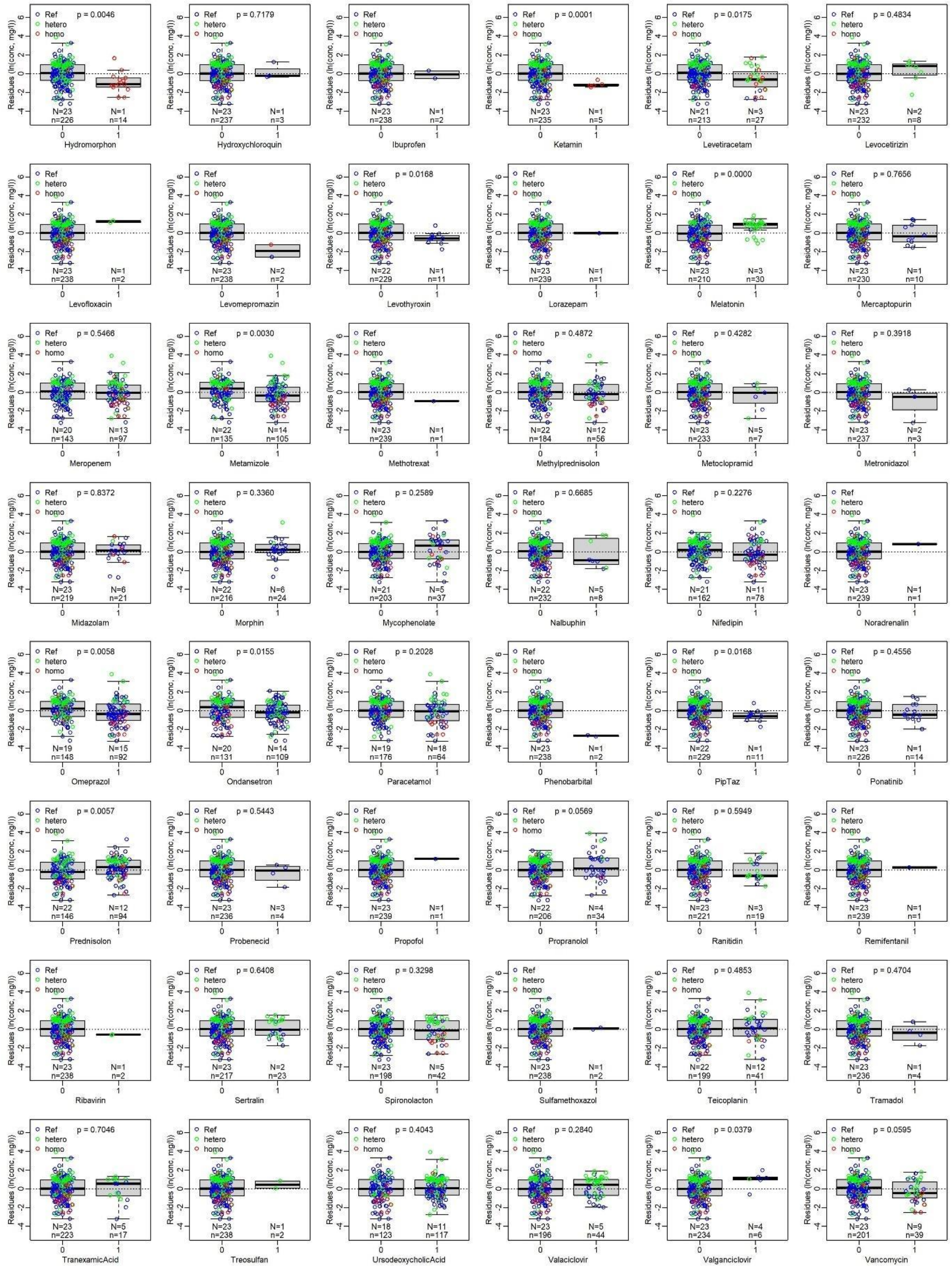

Figure S3. Residues plotted against co-medication as indicated after including dose per body weight and body surface area as covariates in the model. 0, 1 on x-axis, no / with co-medication. *N*, number of patients in a group; *n*, number of C_{trough} measurements in a group; *p*, *p* value of a two-tailed, homoscedastic student's *t*-test. Blue symbols, CYP2C19 rs4244285 GG reference genotype; green symbols, GA; red symbols AA genotype. Plots were prepared for each polymorphic gene and data inspected to identify potential covariates.

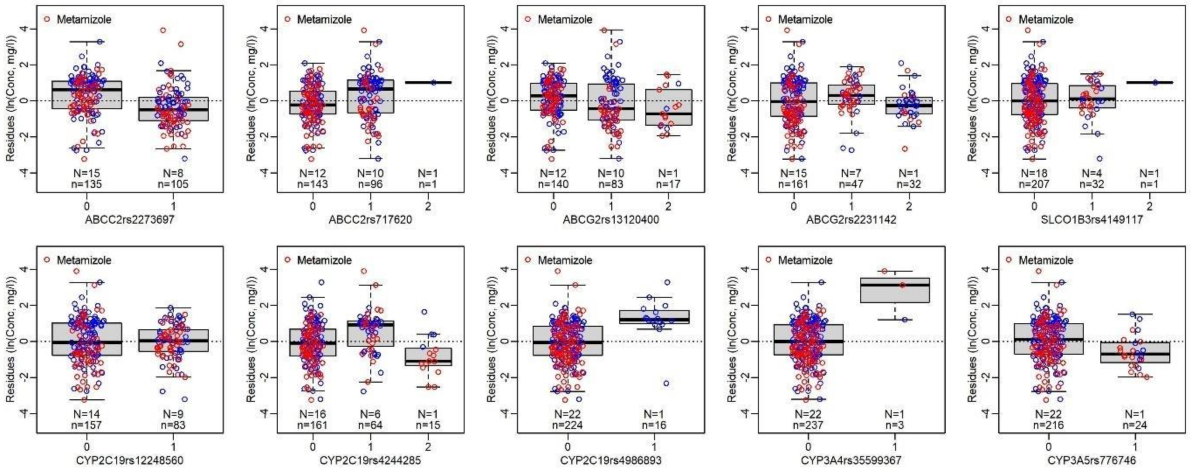

Figure S4. Residues plotted against gene variants as indicated after including dose per body weight and body surface area as covariates in the model. 0, 1, 2 on the x-axis, number of variant alleles in a genotype (0, reference; 1, heterozygous; 2, homozygous for the variant). Blue, without co-medication with the indicated drug (metamizole in the example figure); red, with comedication with the indicated drug. Plots were prepared for each individual co-medicated drug and inspected for potential covariates and finally potential drug×gene interactions.

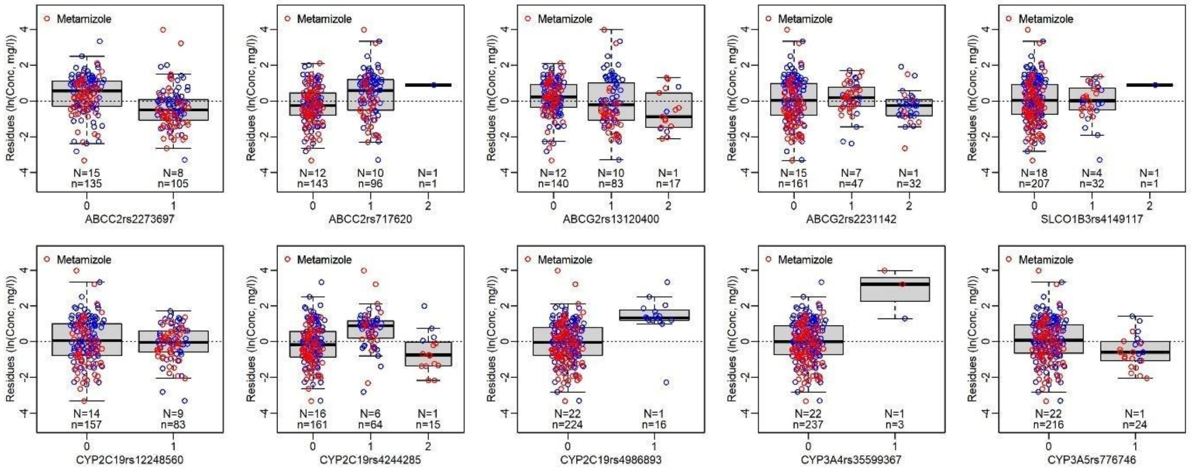

Figure S5. Residues plotted as in Supplementary Figure S4, but including co-medication with ciprofloxacin, levetiracetam and propranolol as covariates.

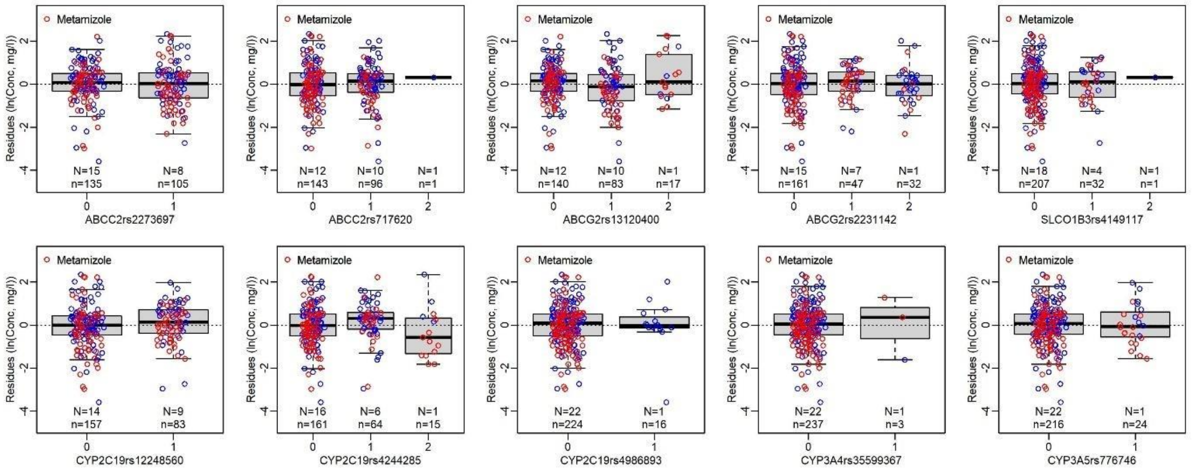

Figure S6. As Supplementary Figure S5, but in addition including the significant effects of the genetic variants in the model.

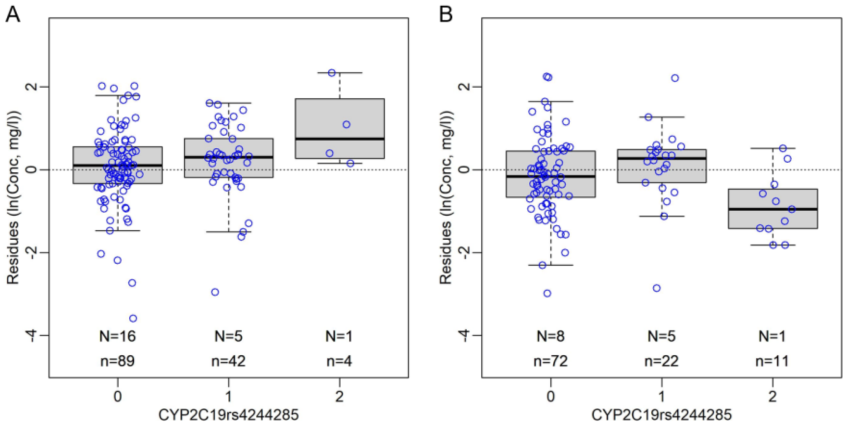

Figure S7. Residues shown in Figure S6 plotted *versus* the CYP2C19 rs4244285 genotype. **A**) C_{trough} without metamizole co-medication. **B**) C_{trough} measured after metamizole co-medication. 0, 1, 2, number of variant alleles. Note that the data for the AA genotype (2 variant alleles) is from the same patient, without and with co-medication with metamizole.

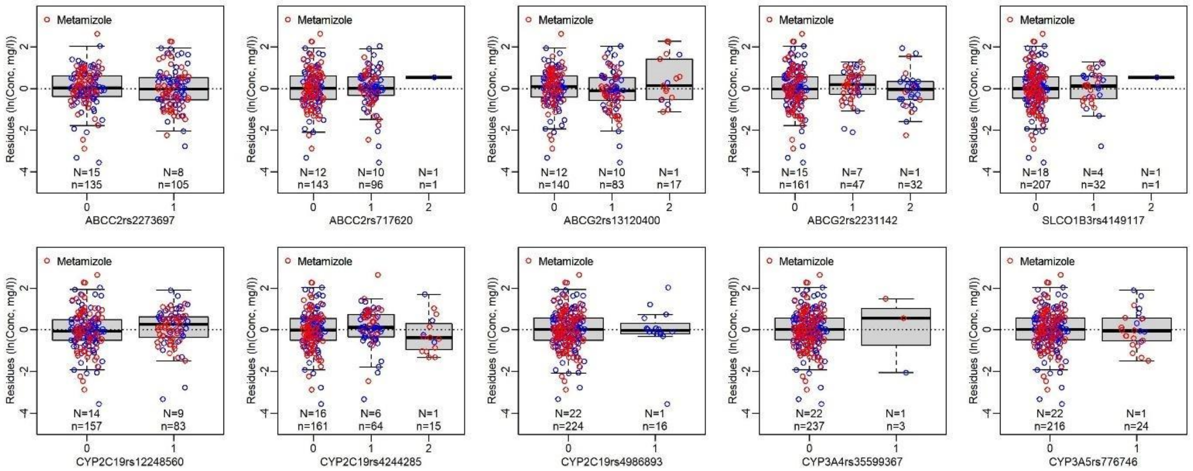

Figure S8. As Supplementary Figure S6, but for the final model, including the interaction term metamizole×CYP2C19 rs4244285 in the model.

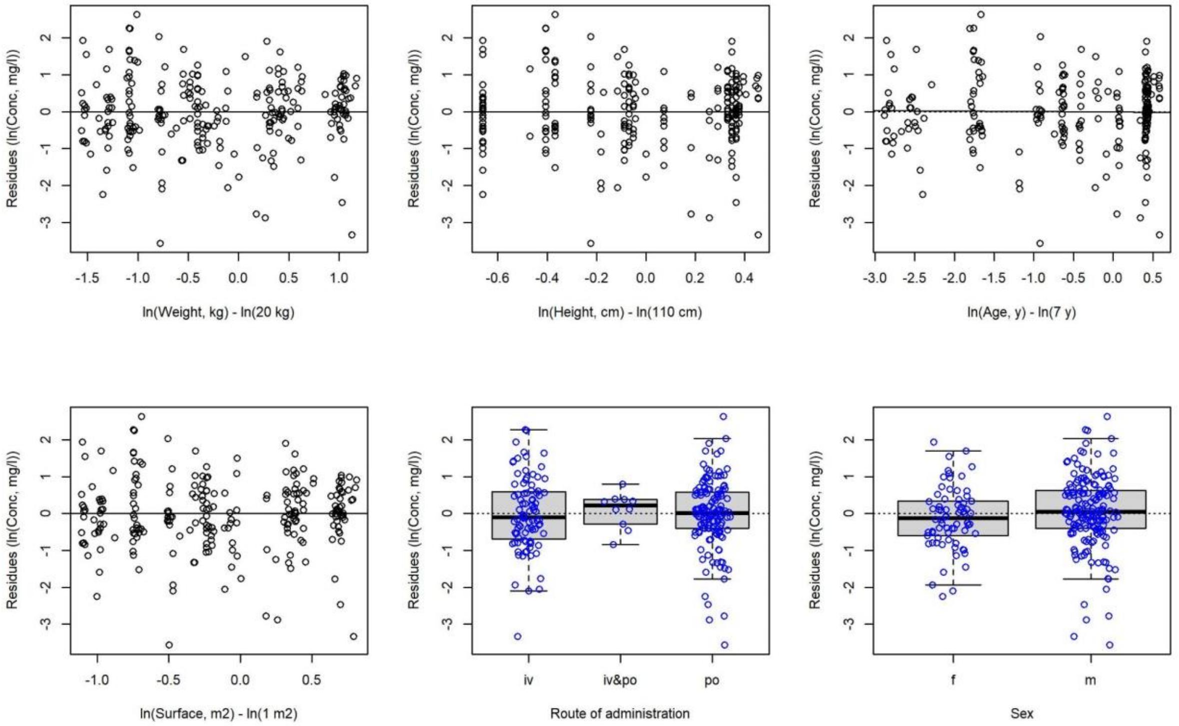

Figure S9. Residues from the final model plotted *versus* demographic parameters.

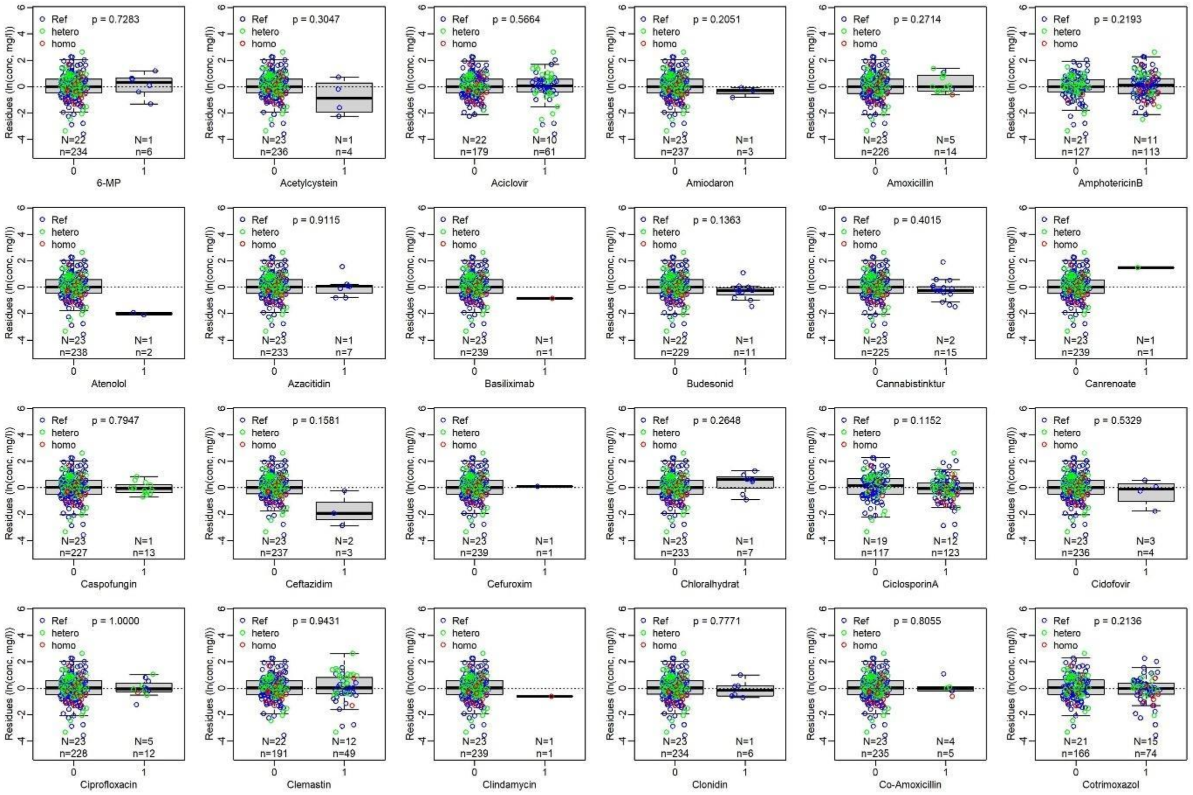

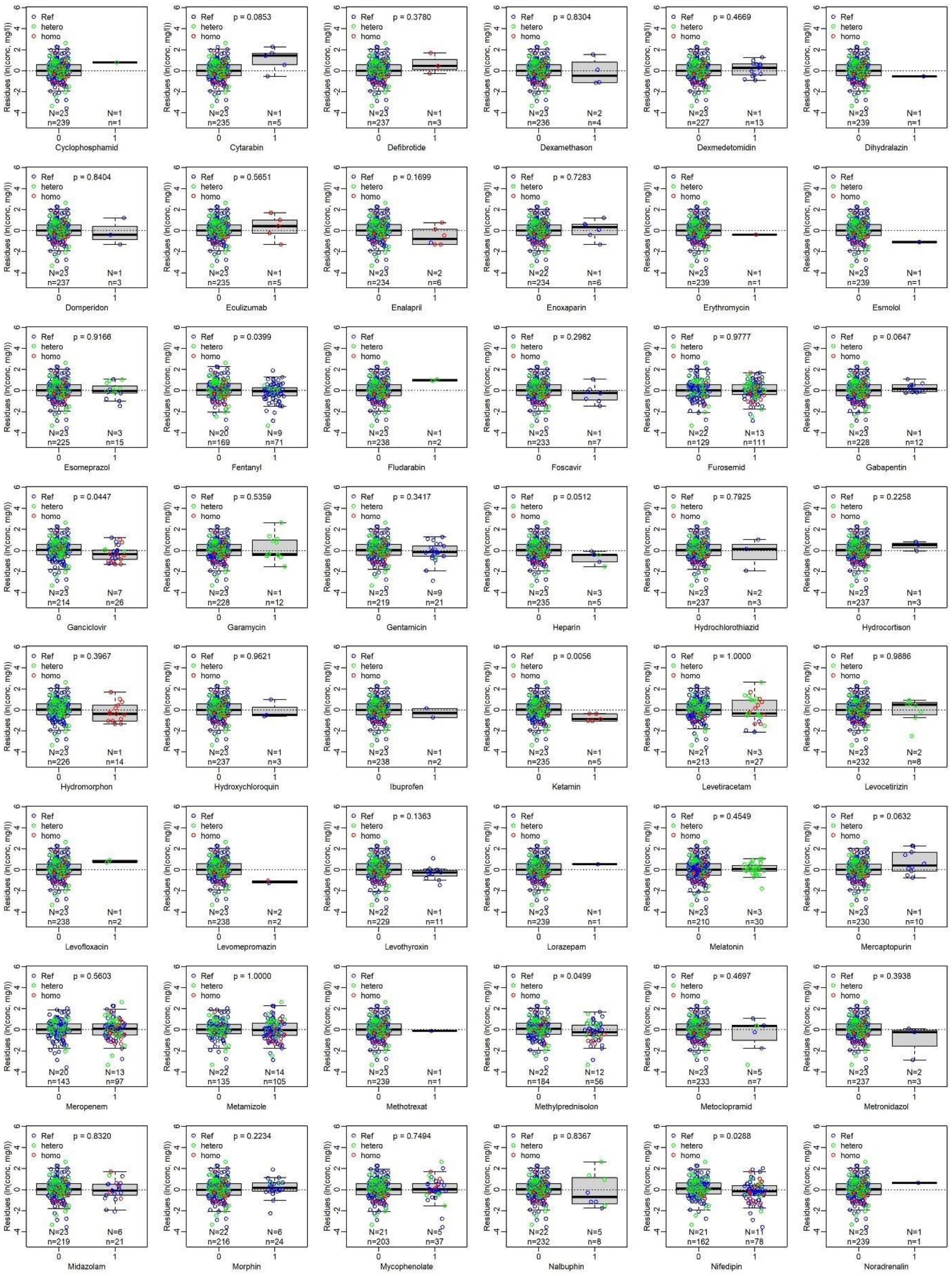
Residues (ln(conc, mg/l))
Ref
hetero
homo
0
1
Cyclophosphamid N=23 n=239 N=1 n=1
Cytarabin p = 0.0853 N=23 n=235 N=1 n=5
Defibrotide p = 0.3780 N=23 n=237 N=1 n=3
Dexamethason p = 0.8304 N=23 n=236 N=2 n=4
Dexmedetomidin p = 0.4669 N=23 n=227 N=1 n=13
Dihydralazin N=23 n=239 N=1 n=1
Domperidon p = 0.8404 N=23 n=237 N=1 n=3
Eculizumab p = 0.5651 N=23 n=235 N=1 n=5
Enalapril p = 0.1699 N=23 n=234 N=2 n=6
Enoxaparin p = 0.7283 N=22 n=234 N=1 n=6
Erythromycin N=23 n=239 N=1 n=1
Esmolol N=23 n=239 N=1 n=1
Esomeprazol p = 0.9166 N=23 n=225 N=3 n=15
Fentanyl p = 0.0399 N=20 n=169 N=9 n=71
Fludarabin N=23 n=238 N=1 n=2
Foscavir p = 0.2982 N=23 n=233 N=1 n=7
Furosemid p = 0.9777 N=22 n=129 N=13 n=111
Gabapentin p = 0.0647 N=23 n=228 N=1 n=12
Ganciclovir p = 0.0447 N=23 n=214 N=7 n=26
Garamycin p = 0.5359 N=23 n=228 N=1 n=12
Gentamicin p = 0.3417 N=23 n=219 N=9 n=21
Heparin p = 0.0512 N=23 n=235 N=3 n=5
Hydrochlorothiazid p = 0.7925 N=23 n=237 N=2 n=3
Hydrocortison p = 0.2258 N=23 n=237 N=1 n=3
Hydromorphon p = 0.3967 N=23 n=226 N=1 n=14
Hydroxychloroquin p = 0.9621 N=23 n=237 N=1 n=3
Ibuprofen N=23 n=238 N=1 n=2
Ketamin p = 0.0056 N=23 n=235 N=1 n=5
Levetiracetam p = 1.0000 N=21 n=213 N=3 n=27
Levocetirizin p = 0.9886 N=23 n=232 N=2 n=8
Levofloxacin N=23 n=238 N=1 n=2
Levomepromazin N=23 n=238 N=2 n=2
Levothyroxin p = 0.1363 N=22 n=229 N=1 n=11
Lorazepam N=23 n=239 N=1 n=1
Melatonin p = 0.4549 N=23 n=210 N=3 n=30
Mercaptopurin p = 0.0632 N=23 n=230 N=1 n=10
Meropenem p = 0.5603 N=20 n=143 N=13 n=97
Metamizole p = 1.0000 N=22 n=135 N=14 n=105
Methotrexat N=23 n=239 N=1 n=1
Methylprednisolon p = 0.0499 N=22 n=184 N=12 n=56
Metoclopramid p = 0.4697 N=23 n=233 N=5 n=7
Metronidazol p = 0.3938 N=23 n=237 N=2 n=3
Midazolam p = 0.8320 N=23 n=219 N=6 n=21
Morphin p = 0.2234 N=22 n=216 N=6 n=24
Mycophenolate p = 0.7494 N=21 n=203 N=5 n=37
Nalbuphin p = 0.8367 N=22 n=232 N=5 n=8
Nifedipin p = 0.0288 N=21 n=162 N=11 n=78
Noradrenalin N=23 n=239 N=1 n=1

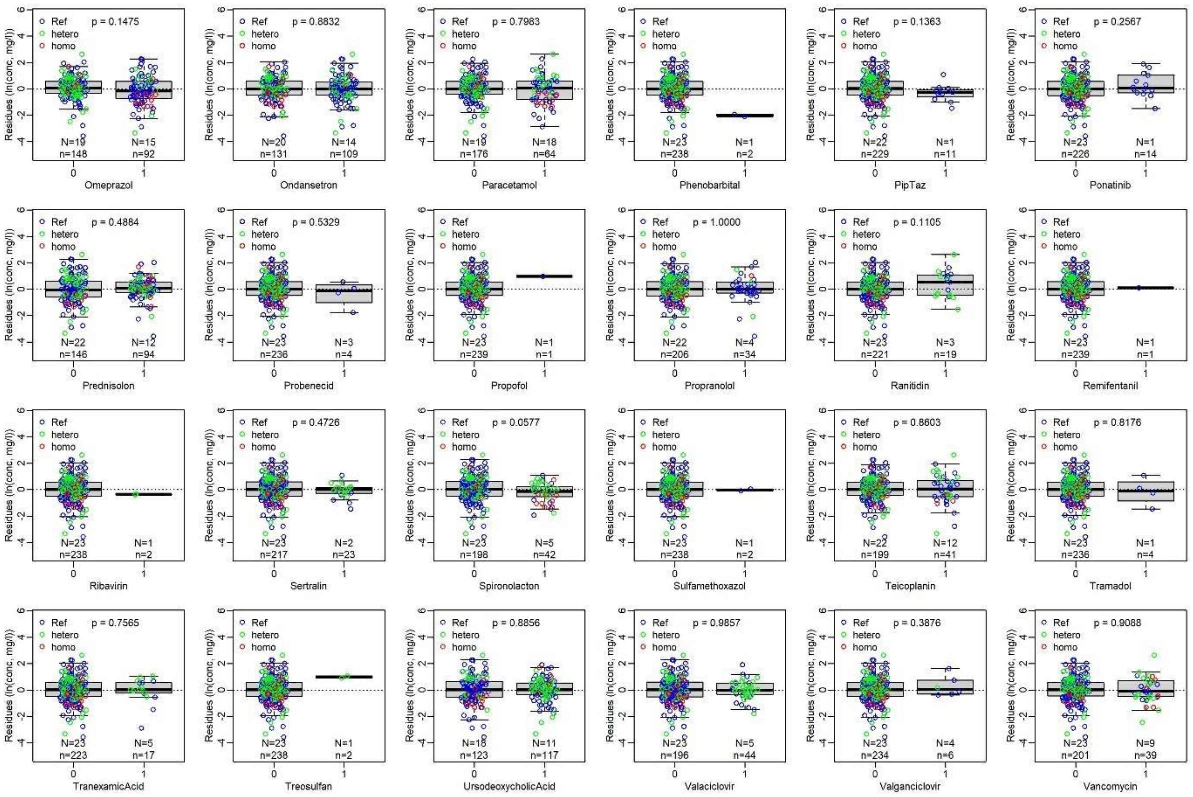

Figure S10. Residues from the final model plotted *versus* co-medication. Colors, as in Supplementary Figure S3.

Table S1. Final model after exclusion of the two carriers of a CYP2C19rs4986893 or CYP3A4rs35599367 variant gene.

**Parameter**	**Reference value for intercept**	**Fit effect**	**SE**	***p***
	**Fixed effects**			
θ_1, Intercept (log(C_{trough}))		-2.6100	0.5293	$<10^{-5}$
θ_2, ln(dose/weight, mg/kg)	n.a.	1.0460	0.2171	$<10^{-5}$
θ_3, Δ ln(surface area, m^2)	ln(1 m^2)	1.2889	0.3049	0.00071
θ_4, ciprofloxacin	No ciprofloxacin	-0.8654	0.3024	0.0047
θ_5, levetiracetam	No levetiracetam	-0.9553	0.3446	0.0084
θ_6, propranolol	No propranolol	-0.5368	0.2883	0.064
θ_7, metamizole	No metamizole	-0.2935	0.1653	0.077
θ_8, ABCC2 rs2273697	θ_8, ABCC2 rs2273697 GG	-0.6082	0.2515	0.042
θ_9, ABCC2 rs717620	θ_9, ABCC2 rs717620 CC	0.5499	0.2625	0.058
θ_{10}, ABCG2 rs2231142	θ_{10}, ABCG2 rs2231142 CC	0.6087	0.2166	0.029
θ_{11}, CYP2C19 rs4244285	θ_{11}, CYP2C19 rs4244285 GG	1.0437	0.2536	0.0010
θ_{14}, metamizole× CYP2C19 rs4244285	No metamizole, CYP2C19 rs4244285 GG	-0.4173	0.2193	0.058
	**Random effects distribution (SD)**			
	ID intercept, 0.3423; Residuals, 0.8782			

Table S2. Final model, but without the metamizole×CYP2C19rs4244285 interaction.

**Parameter**	**Reference value for intercept**	**Fit effect**	**SE**	***p***
		**Fixed effects**		
θ_1, Intercept ($\log(C_{trough})$)		-2.0477	0.4755	$<10^{-4}$
θ_2, ln(dose/weight, mg/kg)	n.a.	0.8566	0.1984	$<10^{-4}$
θ_3, Δ ln(surface area, m^2)	ln(1 m^2)	1.0902	0.2417	$<10^{-4}$
θ_4, ciprofloxacin	No ciprofloxacin	-1.0782	0.2964	0.0034
θ_5, levetiracetam	No levetiracetam	-0.8887	0.2890	0.0023
θ_6, propranolol	No propranolol	-0.5694	0.2733	0.038
θ_8, ABCC2 rs2273697	ABCC2 rs2273697 GG	-0.7527	0.1691	$<10^{-4}$
θ_9, ABCC2 rs717620	ABCC2 rs717620 CC	0.5188	0.1929	0.0077
θ_{10}, ABCG2 rs2231142	ABCG2 rs2231142 CC	0.4543	0.1416	0.0015
θ_{11}, CYP2C19 rs4244285	CYP2C19 rs4244285 GG	0.5802	0.1571	0.00027
θ_{12}, CYP2C19 rs4986893	CYP2C19 rs4986893 GG	1.5350	0.4373	0.00056
θ_{13}, CYP3A4 rs35599367	CYP3A4 rs35599367 CC	3.2609	0.6327	$<10^{-6}$
		**Random effects distribution (SD)**		
		ID intercept, n.a.; Residuals, 0.9628		

n.a., not applicable (see main text)

Table S3. Final model, but without effects for co-medication.

**Parameter**	**Reference value for intercept**	**Fit effect**	**SE**	***p***
		**Fixed effects**		
θ_1, Intercept ($\log(C_{trough})$)		-2.4986	0.5802	$<10^{-4}$
θ_2, ln(dose/weight, mg/kg)	n.a.	0.8581	0.2304	0.00026
θ_3, Δ ln(surface area, m^2)	ln(1 m^2)	1.4067	0.3261	0.00035
θ_8, ABCC2 rs2273697	ABCC2 rs2273697 GG	-0.5329	0.2913	0.094
θ_9, ABCC2 rs717620	ABCC2 rs717620 CC	0.6452	0.3075	0.049
θ_{10}, ABCG2 rs2231142	ABCG2 rs2231142 CC	0.6628	0.2508	0.027
θ_{11}, CYP2C19 rs4244285	CYP2C19 rs4244285 GG	0.5099	0.2458	0.064
θ_{12}, CYP2C19 rs4986893	CYP2C19 rs4986893 GG	1.4170	0.6322	0.0496
θ_{13}, CYP3A4 rs35599367	CYP3A4 rs35599367 CC	2.8894	0.6327	0.0012
		**Random effects distribution (SD)**		
		ID intercept, 0.4501; Residuals, 0.9542		
